# Rice Bran Oil Attenuates Chronic Inflammation by Inducing M2 Macrophage Switching in High-Fat Diet-Fed Obese Mice

**DOI:** 10.3390/foods10020359

**Published:** 2021-02-07

**Authors:** Hyejeong Park, Seungmin Yu, Wooki Kim

**Affiliations:** Department of Food Science and Biotechnology, Graduate School of Biotechnology, Kyung Hee University, Yongin 17104, Korea; 9601ys@naver.com (H.P.); dbtmdals1004@khu.ac.kr (S.Y.)

**Keywords:** rice bran oil, anti-inflammatory, high-fat diet, chronic inflammation, macrophage polarization, obesity

## Abstract

Macrophages are involved in all inflammatory processes from killing pathogens to repairing damaged tissue. In the obese state, macrophages infiltrate into enlarged adipose tissue and polarize into pro-inflammatory M1 macrophages, resulting in chronic low-grade inflammation due to the secretion of inflammatory mediators. Rice bran oil (RBO) is an edible oil containing tocopherols, tocotrienols, and γ-oryzanol. Previous research in normal diet-fed mice suggested that RBO mitigates inflammatory responses by modulating mitochondrial respiration of macrophages. Therefore, we investigated if RBO had an anti-inflammatory effect in diet-induced obese mice by assessing the expression of inflammatory markers in epididymal white adipose tissue (eWAT) and polarization of bone marrow-derived macrophages (BMDMs). Rice bran oil exerted a local anti-inflammatory effect in white adipose tissue by suppressing the production of inflammatory mediators and upregulating transcription of anti-inflammatory genes. Rice bran oil also promoted anti-inflammatory M2 macrophage polarization in BMDMs thereby affecting systemic inflammation. Overall, our in vivo and ex vivo results highlight the potential of RBO as a dietary mediator that can ameliorate obesity-induced chronic low-grade inflammation by mediating the expression of inflammation-related factors and macrophage polarization.

## 1. Introduction

Inflammation is a series of defensive immune responses by the host that enables it to maintain physiological homeostasis by removing stimuli such as microbial infections and tissue injury along with repair of wounded tissues [1]. If the inflammatory milieu persists for some reason without resolving, there is a transition from an acute inflammatory state to a chronic inflammatory state, causing immune system imbalance [2]. Numerous studies have suggested that obesity is associated with a chronic inflammatory state that in turn exacerbates obesity-related inflammation and causes various metabolic disorders. The chronic low-grade inflammation induced by obesity also affects insulin resistance, which can lead to type 2 diabetes [3,4]. Although the exact molecular mechanisms linking obesity and immune changes have not been fully elucidated, activation of adipose tissue residual macrophages and their secretion of inflammatory mediators in adipose tissue have been shown to be downregulated by loss of body weight [5,6].

Macrophages are commonly subcategorized into two groups: M1 and M2. Classically activated M1 macrophages stimulated by toll-like receptor (TLR)ligands and Th1 cytokines, such as lipopolysaccharides (LPS) and interferon (IFN)-γ, respectively, play a key role in initial inflammatory responses by promoting secretion of pro-inflammatory cytokines, producing reactive oxygen species, and engaging in phagocytosis [7,8]. On the contrary, alternatively activated M2 macrophages polarized by Th2 cytokines, namely, interleukin (IL)-4 and IL-13, have an inherent anti-parasite function and are requisite for resolution of inflammation [7,9]. These macrophages can infiltrate into peripheral tissues and are then referred to as tissue-resident macrophages; these macrophages have different phenotypes and functions depending on the environment of the tissue in which they reside [10]. Several studies have shown that macrophages are prone to infiltrating adipose tissues, and adipose tissue-resident macrophages (ATMs) have an inflammatory M1 phenotype in obesity [11]. In the lean state, mice ATMs exhibit increased expression of CD206, arginase-1, and Ym1, which are anti-inflammatory M2 markers, compared to obese mice [12,13].

To further elucidate the metabolic function of macrophages in obesity-induced chronic inflammation, recent studies have profiled the metabolic phenotypes of macrophages in lean and obese states [14]. Specifically, pro-inflammatory M1 macrophages, which are elevated in the obese state, rely on glycolysis and consequent lactate fermentation for their metabolic needs, even in oxygen-rich environments [15]. By contrast, anti-inflammatory M2 macrophages depend on oxidative phosphorylation (OXPHOS) using acetyl-CoA, a consequent product of β-oxidation, revealing a link between inflammation and lipid metabolism [16,17]. In accordance with these findings, several dietary intervention studies have attempted to switch the phenotype of macrophages from an M1 into an anti-inflammatory M2-like phenotype by upregulating OXPHOS through alteration of lipid profiles in the diet [18,19,20].

Rice bran oil (RBO), widely used in salads and frying, is an edible vegetable oil extracted from the hard outer layer of rice grains that contains various phytoceuticals such as sterols, tocopherols, tocotrienols, and γ-oryzanol [21]. Rice bran oil is also known to have a balanced fatty acid composition characterized by high levels of monounsaturated oleic acid (C18:1) and polyunsaturated linoleic acid (C18:2) [22]. Previous studies have demonstrated that RBO not only alleviates hyperglycemia and hyperlipidemia but also exhibits anti-oxidant and immunomodulatory effects [22,23,24,25,26]. Recently, it was demonstrated that RBO exerts anti-inflammatory properties by upregulating the mitochondrial respiration of macrophages in a normal diet-fed mouse model [27], yet the detailed cellular mechanisms and effects of RBO on obesity-induced chronic inflammation are unclear. Therefore, in the current study, we evaluated the effects of RBO on inflammatory markers in epididymal white adipose tissue (eWAT) in vivo and further examined its influence on polarization of bone marrow-derived macrophages (BMDMs) ex vivo, which are involved in systemic immune responses using a high-fat diet mouse model. In parallel to lard in high fat diet, palm oil (PO), a vegetable oil with a high portion of palmitic acid, served as a control.

## 2. Materials and Methods

### 2.1. Animals and Dietary Interventions

The C57BL/6 male mice at the age of 4 weeks were acquired from Raon Bio (Yongin-si, Korea) and acclimated with AIN-76A rodent chow diet for 1 week under a 20 °C climate-controlled dark/light (12 h/12 h) cycle. The fat composition of each experimental diet was modified by replacing lard with rice bran oil (RBO) or palm oil (PO) as shown in Table 1. Commercially available RBO and PO were purchased from Serim Hyunmi (Jungeup-si, Korea) and JCY (Seongnam-si, Korea) [27]. Following 1 week of acclimation, mice were randomly divided into four groups (*n* = 6–8) and received the specified diet and water ad libitum for 10 weeks. Body weight was recorded every week. After dietary interventions, mice were euthanized by CO_2_ inhalation. White adipose tissue was dissected from the epididymis (eWAT), and bone marrow-derived macrophages (BMDMs) were collected from femurs and tibias in an aseptic environment.

### 2.2. RNA Extraction and Quantitative Reverse-Transcription PCR

Total RNA in epididymal white adipose tissue (eWAT) was extracted with FavorPrep™ Tri-RNA reagent (Favorgen, Ping-Tung, Taiwan) and the RNeasy^®^ mini kit (Qiagen, Hilden, Germany) according to the manufacturers’ instructions. The RNA concentration and purity were measured with a Nanodrop 2000 spectrophotometer (Thermo Scientific, Waltham, MA, USA). Expression of adipogenic and pro-/anti-inflammatory genes was assessed by quantitative reverse-transcription PCR (qRT-PCR) using MG One-Step RT-PCR MasterMix (SYBR Green) (MG Med, Seoul, Korea) and a CFX Connect™ Real-Time PCR detection system (Bio-Rad, Hercules, CA, USA). The thermal cycling specifications were 50 °C for 30 min followed by 95 °C for 10 min and then 40 cycles of 95 °C for 5 s and 60 °C for 40 s. Reactions were performed in a 20 μL reaction volume. Primers used are listed in Table 2, and relative gene transcription levels were calculated by normalizing the threshold cycle (Ct) values of each gene to that of the internal reference gene, β-actin, using the delta–delta Ct method (2^−ΔΔCt^).

### 2.3. Culture and Polarization of Bone Marrow-Derived Macrophages (BMDMs) 

For a separation of mononuclear cells in femurs and tibias of C57BL/6 mice, red blood cells (RBCs) were removed by 1× RBC lysis buffer (eBioscience, San Diego, CA, USA) on ice for 10 min. Following removal of RBC, mononuclear cells were differentiated into bone marrow-derived macrophages (M0 BMDMs) with 10 ng/mL macrophage colony-stimulating factor (M-CSF, Sigma–Aldrich, St. Louis, MO, USA) for 7 days. The BMDMs were further cultured in Iscove’s modified Dulbecco’s medium (IMDM) supplemented with 10% FBS at 37 °C in a 5% CO_2_ incubator. Mature BMDMs were treated with either 100 ng/mL LPS and 50 ng/mL IFN-γ, or 10 ng/mL IL-4 and 10 ng/mL IL-13 for 24 h to induce M1 or M2 macrophage polarization, respectively. 

### 2.4. Assessment of Macrophage Activation Markers

Expression of surface activation markers on BMDMs was quantified by flow cytometric analysis following staining with specific antibodies conjugated with fluorescent molecules. Aforementioned BMDMs were rinsed with cold PBS containing 2% FBS and incubated with mouse CD16/CD32-specific mAbs (Fc block, eBioscience) for 15 min at 4 °C to inhibit non-specific bindings. Cells were then stained with fluorescence-conjugated antibodies as follows: fluorescein isothiocyanate-conjugated anti-mouse F4/80 (F4/80-FITC, eBioscience), anti-mouse CD11b conjugated with peridinin-chlorophyll protein-cyanin5.5 (CD11b-PerCP-Cy5.5, eBioscience), allophycocyanin-conjugated anti-mouse CD11c (CD11c-APC, eBioscience), and phycoerythrin-conjugated anti-mouse CD206 (CD206-PE, eBioscience) for 10 min at 4 °C, simultaneously. Cells were then washed twice and resuspended in ice-cold PBS followed by analysis using a flow cytometer (Accuri™ C6, BD Bioscience, San Jose, CA, USA). The double positive BMDMs for F4/80 and CD11b were gated as mature macrophages. Relative expression of activation markers was determined by mean fluorescence intensity (MFI) and normalized to the MFI of the normal died-fed control using FlowJo^®^ software (BD Bioscience).

### 2.5. Cytokine Quantification

Pro-inflammatory (IL-6 and tumor necrosis factor (TNF)-α) and anti-inflammatory (IL-10) cytokines in M1 and M2 BMDM culture medium were quantified with BD OptEIA™ mouse enzyme-linked immunosorbent assay (ELISA) kits (BD Bioscience) following the manufacturer’s instructions. Briefly, specific capture antibodies were added to 96-well plates and plates were incubated overnight at 4 °C. Then, excess antibodies were "rinsed off and assay diluent was added to wells to inhibit non-specific protein binding. Bone marrow-derived macrophage culture supernatants and purified cytokine standards were added to wells at designated concentrations followed by a 2 h incubation at room temperature. Following incubation and washing steps, plate-bound cytokines were incubated with biotin-conjugated detection antibodies in the presence of streptavidin–horseradish peroxidase (SAv-HRP) for 1 h. After a series of washing steps, tetramethylbenzidine (TMB) substrate solution was added to each well followed by a 30 min incubation in the dark. Finally, stop solution (1 M H_3_PO_4_) was applied, and absorbance at a wavelength of 450 nm was measured using a microplate reader (Bio-Rad).

### 2.6. Statistical Analysis

All experimental data are expressed as means ± standard errors of the mean (SEM). Statistical significance of differences among experimental groups were analyzed by one-way analysis of variance (ANOVA) followed by a post-hoc Tukey’s multiple comparison test using PRISM software (GraphPad Software, La Jolla, CA, USA). Significant differences are indicated with different letters, and a *p*-value less than 0.05 was considered statistically significant.

### 2.7. Ethics Statement

The animal protocols in this work were evaluated and approved by the Institutional Animal Care and Use Committee (IACUC) of Kyung Hee University (Approval ID: KHGASP-19-134).

## 3. Results

### 3.1. Body Weight Gain Was Suppressed by RBO in High-Fat Diet-Fed C57BL/6 Mice

**Figure 1:**
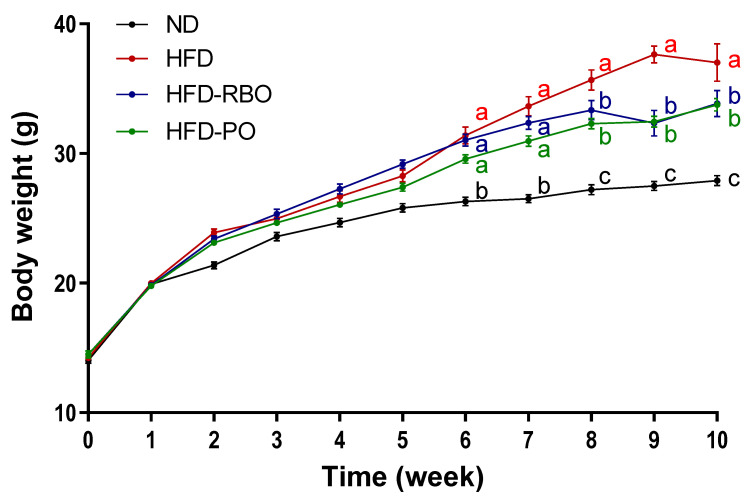
Body weight changes in C57BL/6 male mice who were allowed ad libitum access to the indicated diets as pellets for 10 weeks. Results are reported as means ± SEM. Mean values with different letters are significantly different within the specific week (*p* < 0.05). (ND, normal diet; HFD, high fat diet; HFD-RBO, high-fat diet-rice bran oil; HFD-PO, high-fat diet-palm oil).

Four-week-old C57BL/6 male mice were fed modified high-fat diets where the lard was replaced with edible rice bran oil or palm oil, and weight gain of these mice was compared to that of control mice fed the AIN-76A normal diet or high-fat diet. Mice were fed either high-fat diet-rice bran oil (HFD-RBO), high-fat diet-palm oil (HFD-PO), the control high-fat diet (HFD), or normal AIN-76A (ND) diet for 10 weeks ad libitum. The food intake was assessed for each week, and there were no significant differences among dietary groups (data not shown). After 6 weeks of feeding, there were significant differences in body weights between the normal diet-fed group (ND: 25.81 ± 0.33 g) and high-fat diet-fed groups (HFD: 28.26 ± 0.45 g, HFD-RBO: 29.17 ± 0.32 g and HFD-PO: 27.39 ± 0.31 g). Following 8 weeks of feeding, HFD-RBO (32.37 ± 0.5 g) and HFD-PO (31 ± 0.4 g)-fed groups had significantly lower body weights than HFD (33.64 ± 0.74 g)-fed control mice, and the decrease in body weight gain relative to the HFD control group was maintained until 10 weeks (ND: 27.5 ± 0.35, HFD: 37.64 ± 0.65, HFD-RBO: 32.35 ± 0.1, and HFD-PO: 32.46 ± 0.41 g) (Figure 1).

### 3.2. RBO and PO Were Associated with Downregulation of Adipogenesis in Mice Epididymal White Adipose Tissue (eWAT)

**Figure 2:**
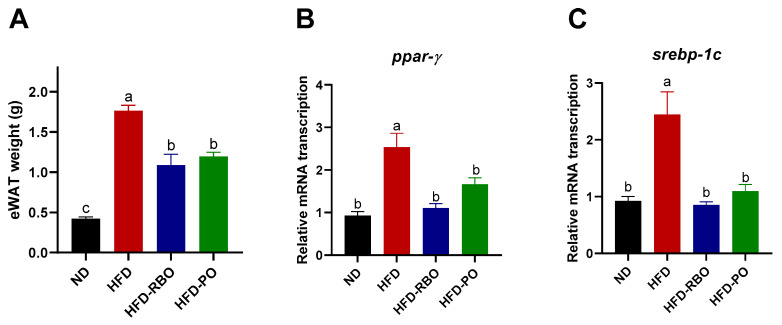
Epididymal white adipose tissue (eWAT) weights and expression of adipogenic genes. (**A**) Epididymal white adipose tissue was dissected and its weight measured after 10 weeks of dietary intervention. The expression of adipogenic genes, (**B**) *ppar-γ* and (**C**) *srebp-1c*, was assessed by qRT-PCR. Relative transcription levels of the target gene were calculated by normalization to β-actin using the delta–delta Ct method (2^−ΔΔCt^). Data are expressed as means ± SEM. Different letters indicate significant differences between groups (*p* < 0.05) (ND, normal diet; HFD, high fat diet; HFD-RBO, high-fat diet-rice bran oil; HFD-PO, high-fat diet-palm oil).

To investigate the relevance of body weight gain inhibition by edible oil (RBO and PO) intervention, the weight of epididymal white adipose tissue and transcription of adipogenic genes were examined. High-fat diet-fed groups exhibited a significant increase in eWAT weight compared to the normal diet-fed group, but HFD-RBO and HFD-PO-fed groups showed a significantly lower increase in eWAT weight than the HFD-fed group (ND: 0.42 ± 0.02, HFD: 1.76 ± 0.07, HFD-RBO: 1.09 ± 0.13 and HFD-PO: 1.2 ± 0.05 g) (Figure 2A). Similar results were observed for the transcription of mRNAs related to lipogenesis in adipose tissue. Transcript levels of peroxisome proliferator-activated receptor (*ppar)-γ* and sterol regulatory element-binding protein (*srebp)-1c* were significantly downregulated in HFD-RBO and HFD-PO-fed mice compared to HFD-fed obese mice, but there were no significant differences in expression of these genes between HFD-RBO and HFD-PO-fed mice and ND-fed mice (Figure 2B,C). Consequently, the suppressed body weight gain in response to consumption of RBO or PO may be associated with a decrement in eWAT weight through downregulation of adipogenic gene expression in high-fat diet-fed mice.

### 3.3. Rice Bran Oil Induces the Expression of M2-Macrophage Markers in the eWAT of HFD-Fed Mice

**Figure 3:**
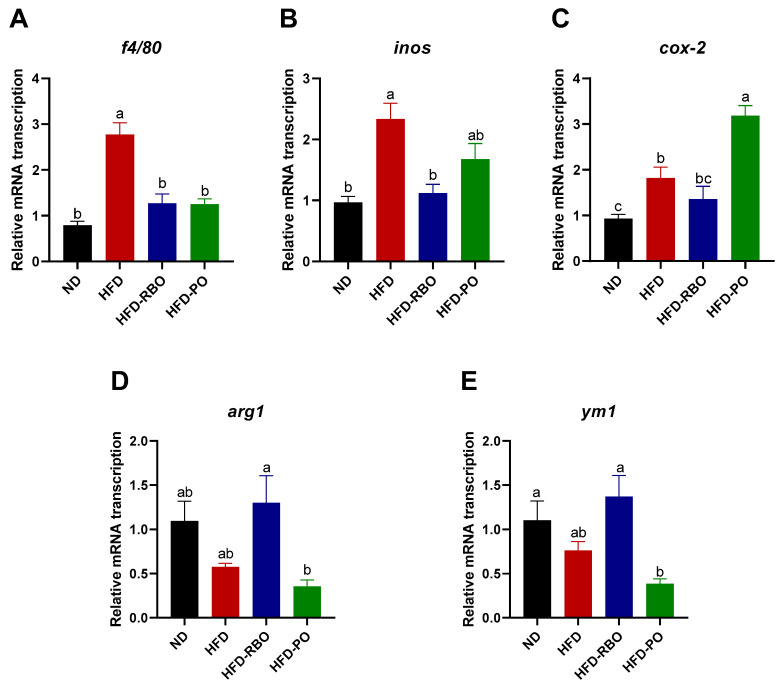
Analyses of mRNA transcription of macrophage polarization in eWAT by qRT-PCR. The mRNA transcription of macrophage marker (**A**) *f4/80*, the M1 markers (**B**) *inos* and (**C**) *cox-2*, and the M2 markers (**D**) *arg1* and (**E**) *ym1* in eWAT. Results were normalized to internal control β-actin gene by using the delta–delta Ct method (2^−ΔΔCt^). Data are presented as the mean ± SEM, and significant differences are indicated with different letters (*p* < 0.05). (ND, normal diet; HFD, high fat diet; HFD-RBO, high-fat diet-rice bran oil; HFD-PO, high-fat diet-palm oil).

Macrophages can infiltrate into adipose tissue and be polarized to different phenotypes that have different immune functions. Obesity promotes macrophage accumulation, proliferation, and polarization to classically activated or pro-inflammatory M1 macrophages, which contributes to local inflammation in adipose tissues. To further investigate the correlation between weight loss and immune responses in high-fat diet-fed obesity-induced mice, the transcription of macrophage polarization-related mRNAs in eWAT was assessed. A significant increase in the macrophage marker f4/80 was observed in the HFD-fed group, but there were no significant differences in expression of this factor among the other groups (Figure 3A). With regard to pro-inflammatory M1 markers, RBO significantly suppressed inducible nitric oxide synthase (*inos*) transcription compared to that in the other high-fat diet-fed groups (Figure 3B). A similar tendency was observed for cyclooxygenase (*cox-2*) mRNA level in mice that received RBO, but no significant differences were noted between the HFD-RBO-fed group and the ND- and HFD-fed groups. Contrary to the *inos* findings, HFD-PO-fed mice exhibited significantly upregulated transcription of *cox-2* compared to the other diet-fed mice groups (Figure 3C). With regard to the expression of anti-inflammatory M2 markers, the HFD-RBO-fed group exhibited upregulated arginase 1 (*arg1*) and chitinase-like proteins, *ym1* transcription compared to the other groups, with a significant difference in expression of these markers compared to the HFD-PO-fed group (Figure 3D,E). These results indicate that rice bran oil may ameliorate local inflammation and contribute to anti-inflammatory macrophage polarization in the white adipose tissue of high-fat diet-induced obese mice.

### 3.4. Rice Bran Oil Increases Surface M2 Marker Expression in Mouse BMDMs

**Figure 4:**
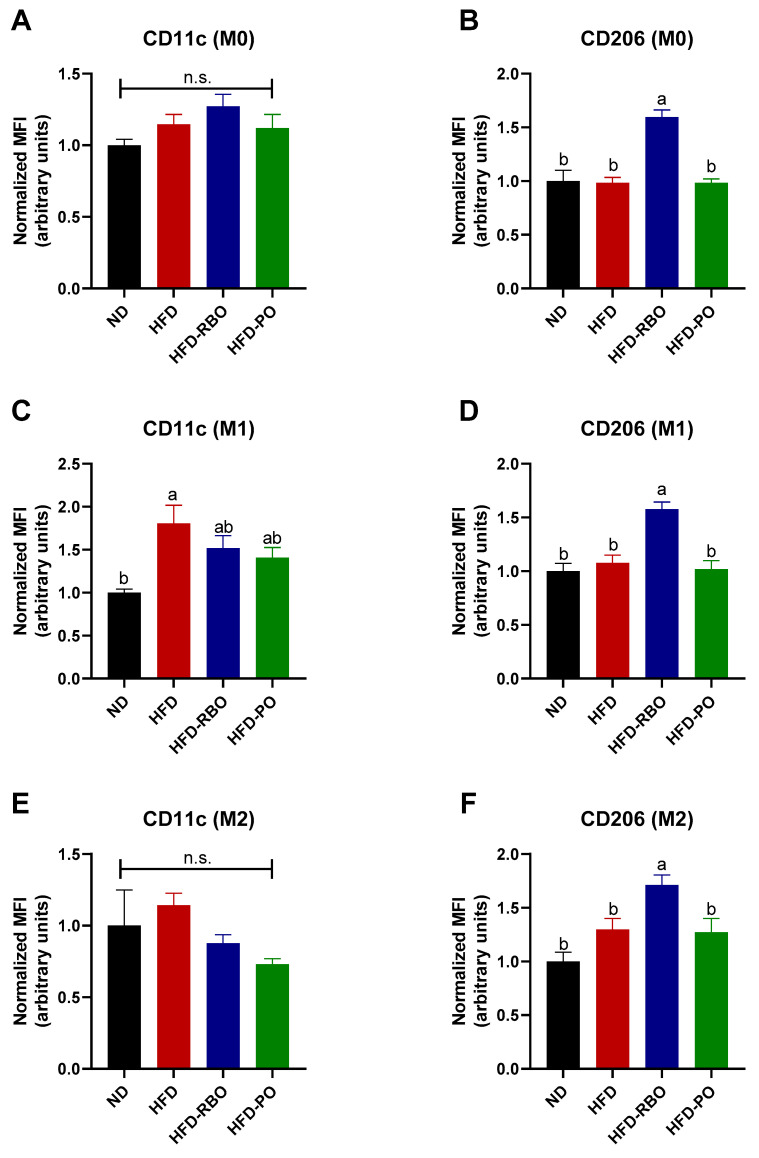
Expression of macrophage polarization markers in BMDMs. The BMDMs were stained with specific antibodies and mean fluorescence intensity (MFI) was assessed by flow cytometry. The F4/80^+^CD11b^+^ population cells were gated and designated as macrophages. The expression of (**A**,**C**,**E**) CD11c as an M1 marker and (**B**,**D**,**F**) CD206 as an M2 marker was further assessed in non-polarized (M0), polarized M1 BMDMs, and M2 BMDMs. Results were normalized to the MFI of the ND-fed control. n.s. no significance. Values are expressed as means ± SEM, and different letters indicate statistical significance (*p* < 0.05) (ND, normal diet; HFD, high fat diet; HFD-RBO, high-fat diet-rice bran oil; HFD-PO, high-fat diet-palm oil).

Given that the phenotype of adipose tissue-resident macrophages was modulated towards an anti-inflammatory M2 macrophage phenotype by rice bran oil, we further examined whether rice bran oil affects systemic inflammation by changing BMDMs ex vivo. Bone marrow-derived macrophages isolated from normal diet and custom high-fat diet-fed mice were polarized into either M1 or M2 phenotypes by addition of LPS/IFN-γ and IL-4/IL-13 to the culture medium, respectively. After polarization, the expression of the M1 macrophage surface marker, CD11c, and the M2 macrophage surface marker, CD206, was assessed by specific staining with fluorescence-conjugated antibodies followed by flow cytometry. In non-polarized (M0) cells, the expression of CD11c was similar between the normal diet and high-fat diet-fed groups (ND: 1.0 ± 0.04, HFD: 1.15 ± 0.07, HFD-RBO: 1.28 ± 0.08, and HFD-PO: 1.12 ± 0.1) (Figure 4A), whereas rice bran oil significantly increased the expression of CD206 (1.6 ± 0.07) compared to the other diet-fed groups (ND: 1.0 ± 0.1, HFD: 0.98 ± 0.05, HFD-PO: 0.98 ± 0.04) (Figure 4B). In M1-induced BMDMs, CD11c expression was significantly upregulated in the HFD-fed group (1.81 ± 0.21) than the normal diet-fed control (1.00 ± 0.04). The HFD-RBO (1.52 ± 0.15) and HFD-PO-fed (1.41 ± 0.12) groups exhibited similar CD11c expression levels compared to both ND and HFD-fed groups (Figure 4C). Similar to the M0 results, rice bran oil significantly upregulated CD206 expression (1.58 ± 0.06) in M1-induced cells compared to the other diet types (ND: 1.0 ± 0.07, HFD: 1.08 ± 0.07, and HFD-PO: 1.02 ± 0.08) (Figure 4D). In the IL-4/IL-13-stimulated condition (M2), the HFD-RBO (0.88 ± 0.06) and HFD-PO (0.73 ± 0.04) groups showed decreased expression of CD11c, but this decreased expression was not statistically significant compared to the other diet groups (ND: 1.0 ± 0.25 and HFD: 1.14 ± 0.08) (Figure 4E). Interestingly, rice bran oil also significantly increased CD206 expression (1.71 ± 0.09) in M2 BMDMs compared to other diets (ND: 1.0 ± 0.09, HFD: 1.3 ± 0.1 and HFD-PO 1.27 ± 0.13) as in the M0- and M1-induced conditions (Figure 4F). These results indicate that rice bran oil increases the expression of anti-inflammatory activation markers and induces M2 polarization of non-polarized and polarized subsets of BMDMs, which mediate systemic inflammation.

### 3.5. Rice Bran Oil Regulates Cytokine Production in M1 and M2 BMDMs

**Figure 5:**
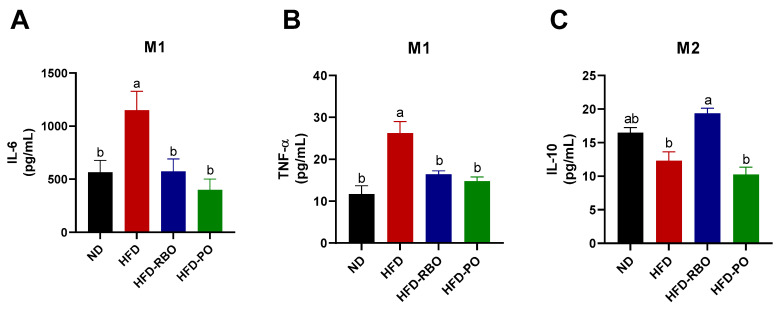
Cytokine expressions in M1- and M2-induced BMDMs. Levels of cytokines in polarized BMDM culture supernatants were determined by ELISA. Secretion of the pro-inflammatory cytokines (**A**) IL-6 and (**B**) TNF-α was assessed under LPS/IFN-γ-stimulated M1 conditions. Levels of the anti-inflammatory cytokine (**C**) IL-10 were quantified in IL-4/IL-13-induced M2 BMDMs. Data are presented as means ± SEM. Significant differences are indicated with different letters (*p* < 0.05) (ND, normal diet; HFD, high fat diet; HFD-RBO, high-fat diet-rice bran oil; HFD-PO, high-fat diet-palm oil).

Following the observation of the anti-inflammatory (M2-like) effects of rice bran oil on BMDM polarization, we further examined the secretion of pro-/anti-inflammatory cytokines in the supernatants of M1 or M2 polarized BMDMs ex vivo. Under LPS/IFN-γ stimulating conditions (M1), the secretion of the pro-inflammatory cytokine IL-6 was significantly suppressed by HFD-RBO (573.7 ± 117.9 pg/mL) and HFD-PO (399.5 ± 102.2 pg/mL) intervention, while the HFD-fed group (1,149 ± 179 pg/mL) exhibited significantly increased IL-6 production compared to the normal diet-fed control (565.4 ± 113 pg/mL) (Figure 5A). The TNF-α production showed a similar pattern in M1 BMDMs (ND: 11.68 ± 2.02 pg/mL, HFD: 26.22 ± 2.76 pg/mL, HFD-RBO: 16.40 ± 0.86 pg/mL, and HFD-PO: 14.74 ± 1.05 pg/mL) (Figure 5B). In contrast, rice bran oil significantly upregulated the expression of the anti-inflammatory cytokine IL-10 (19.36 ± 0.77 pg/mL) compared to other high-fat diet-fed groups (HFD: 12.3 ± 1.34 pg/mL and HFD-PO: 10.26 ± 1.08 pg/mL), but there was no significant difference in IL-10 expression between HFD-RBO and normal diet-fed control (16.46 ± 0.8 pg/mL) M2-induced BMDMs (Figure 5C). These data suggest that rice bran oil regulates systemic inflammation by downregulating the expression of inflammatory cytokines and elevating the expression of anti-inflammatory cytokines in M1 or M2 polarized BMDMs.

## 4. Discussion

Inflammation is a complex immune response of the body to injury [28]. Inflammatory response starts as an acute reaction, whereas unresolved immune activation causes chronic inflammation by affecting local tissues or systemic immunity [29]. The central role of innate immune cells in the inflammatory milieu is to distinguish self from non-self substances to boost host–defense responses [30]. As the major innate immune cells, macrophages play critical roles not only as phagocytic cells but also as regulatory cells [31]. In this regard, macrophages are classified as classically activated M1 macrophages or alternatively activated M2 macrophages based on their different functional phenotypes. M1 macrophages polarized by LPS or interferon (IFN)-γ produce pro-inflammatory mediators, such as IL-6 and TNF-α, that contribute to inflammatory, microbicidal, and tumoricidal activities. By contrast, M2 macrophages that differentiate in response to IL-4 or IL-13 stimulation release anti-inflammatory factors, such as IL-10, and have inherent anti-parasitic, wound healing, and tissue repair properties [9,32].

Chronic overnutrition and resultant obesity are associated with metabolic dysfunction such as type 2 diabetes and insulin resistance. Numerous studies have demonstrated that obesity also causes chronic low-grade inflammation in adipose tissues. In obese adipose tissue, there is increased infiltration of macrophages and polarization of residual macrophages into inflammatory M1 macrophages [33]. Cytokines released by M1 macrophages or adipocytes stimulate adipogenesis and consequent inflammatory responses. By contrast, residual M2 macrophages maintain a lean state through regulation of tissue homeostasis and inflammatory responses [34,35].

Regulation of chronic inflammation through switching the macrophage phenotype using dietary components is of growing interest. A prior study demonstrated that rice bran oil (RBO) had anti-inflammatory effects by regulating mitochondrial respiration of macrophages in a normal diet-fed mice model [27]. Therefore, in the current study, we evaluated the anti-inflammatory effects of rice bran oil in a high-fat diet-induced obese mouse model by performing in vivo and ex vivo experiments. High-fat diet (HFD) containing lard with a high content of palmitic acid served as the positive control, and a diet in which the lard in the HFD was replaced with vegetable palm oil, which has a similar fatty acid composition to lard, was used as another control (HFD-PO). Mice fed the AIN-76A diet, which contains corn oil, served as the negative control.

To investigate whether RBO suppressed local inflammation in our high-fat diet-induced obese model, we monitored weight gain in the different mouse groups over a 10-week period. The HFD-fed mice gained significantly more body weight than normal diet-fed mice, but those mice that received RBO or PO had less of a weight increase than HFD-fed mice (Figure 1). Consistent with these findings, the weight of eWAT was significantly reduced in the RBO- and PO-groups than in the high-fat diet control group (Figure 2A). 

Peroxisome proliferator-activated receptor (*ppar)-γ* and sterol regulatory element-binding protein (*srebp)-1c* are key regulatory factors in lipid metabolism and adipocyte differentiation [36,37]. Several studies have demonstrated that transcript levels of PPAR-γ and SREBP-1c are increased in high-fat induced obese mice [38,39], consistent with our findings in the current study (Figure 2B,C). Rice bran oil and PO also significantly downregulated transcript levels of *ppar-γ* and *srebp-1c* after high-fat dietary intervention compared to the HFD-fed control. These results imply that downregulation of adipogenic genes by RBO and PO results in a reduction in eWAT weight and consequent suppression of body weight gain despite consumption of a high-fat diet.

To further investigate the effects of RBO and PO on high-fat diet-induced chronic inflammation in local eWAT, we assessed the expression of surface activation markers of infiltrated or accumulated macrophages in eWAT. As shown in Figure 3, expression of *f4/80*, a macrophage marker, was significantly suppressed by RBO- and PO-HFDs compared to a regular HFD, indicating that the population of adipose tissue residual macrophages was decreased by RBO and PO to a level similar to that seen in the normal diet control. Expression of the pro-inflammatory marker *inos* was significantly downregulated by HFD-RBO compared to HFD and HFD-PO intervention, but although *cox-2* transcription was decreased by HFD-RBO treatment, this difference was not statistically significant compared to expression of *cox-2* in the HFD and ND control groups. Interestingly, HFD-PO increased *cox-2* transcription compared to other diets. The RBO-HFD significantly elevated transcript expression of the anti-inflammatory markers *arg1* and *ym1* to a level similar to that of the normal diet control group. These data suggest that RBO can ameliorate obesity-induced chronic inflammation in local adipose tissue.

Following investigation of pro-/anti-inflammation marker expression in eWAT, we investigated the effects of RBO on polarization of macrophages. Bone marrow-derived macrophages were collected from sacrificed mouse femurs and tibias and then polarized into M1 or M2 macrophages in LPS/IFN-γ and IL-4/IL-13 microenvironments, respectively. As shown in Figure 4, RBO significantly enhanced the expression of CD206, a representative activation marker of anti-inflammatory M2 macrophages, in all subsets of macrophages (M0, M1, and M2) to a greater extent than observed for subsets of macrophages from the normal diet and other high-fat diet-fed groups. Furthermore, RBO significantly inhibited the secretion of the pro-inflammatory cytokines IL-6 and TNF-α while inducing increased expression of the anti-inflammatory cytokine IL-10 compared to the HFD control (Figure 5). Taken together, these data indicate that increased energy expenditure by modified lipid components accelerates anti-inflammatory M2-like phenotype changes of macrophages. These results also suggest that RBO can promote M2 polarization of macrophages in the obese state. 

Interestingly, PO did not affect the expression of macrophage polarization markers but significantly reduced IL-6 and TNF-α production in the inflammatory M1 microenvironment (Figure 4 and Figure 5). In this regard, several previous studies have demonstrated that palm oil has anti-inflammatory effects [40,41,42,43]. Those studies focused on specific components of palm oil, such as δ-tocotrienol, whereas we used whole palm oil in our high-fat diet-induced obese mice model. Therefore, additional studies are required to fully elucidate differences between PO and RBO on inflammatory gene transcription in eWAT and the expression of the surface proteins and cytokines in BMDMs.

Rice bran oil has previously been shown to attenuate obesity, hyperlipidemia, and inflammation in mice and rats [25,26,44]. A previous study also demonstrated that RBO exerted its anti-inflammatory effects through modulating the mitochondrial respiration of macrophages in normal diet-fed mice [27]. In this regard, there is a lack of data regarding the effects of palm oil on local chronic inflammation and macrophage polarization in high-fat diet-induced obese mouse models. The results of the current study suggest that RBO has potent regulatory effects on obesity-induced chronic inflammation in local adipose tissues as well as systemic immune responses by modulating macrophage polarization and lipid metabolism. However, to validate our in vivo and ex vivo results, involved signaling pathways need to be identified and the precise nature of the interactions between adipocytes and macrophages requires further investigation.

## Figures and Tables

**Table 1:** Experimental diet composition.

Ingredient (g/kg)	Normal Diet	High-Fat Diet	High-At Diet-RBO	High-Fat Diet-PO
Casein	200	200	200	200
DL-methionine	3	3	3	3
Corn starch	150	111	111	111
Sucrose	500	370	370	370
Cellulose	50	50	50	50
Corn Oil	50	30	30	30
Lard	-	170	-	-
Rice bran oil	-	-	170	-
Palm oil	-	-	-	170
Mineral mix S10001	35	42	42	42
Vitamin mix V1001	10	12	12	12
Choline Bitartrate	2	2	2	2

**Table 2:** Sequences of primers used for qRT-PCR.

Gene	Primer Sequence
*β-actin*	Forward, AGGCCCAGAGCAAGAGAG Reverse, GGGTGTTGAAGGTCTCAAAC
*p* *par-γ*	Forward, TTTTCAAGGGTGCCAGTTTCAATCC Reverse, AATCCTTGGCCCTCTGAGAT
*srebp-1c*	Forward, AATGGTCCAGGCAAGTTCGT Reverse, TCCCTCTCAGCTGTGGTGGTGAA
*f4/80*	Forward, AAAGACTGGATTCTGGGAAGTTTGG Reverse, CGAGAGTGTTGTGGCAGGTTG
*inos*	Forward, CAGAGGACCCAGAGACAAGC Reverse, TGCTGAAACATTTCCTGTGC
*cox-2*	Forward, TTCAAAAGAAGTGCTGGAAAAGGT Reverse, GATCATCTCTACCTGAGTGTCTTT
*arg1*	Forward, CTGGCAGTTGGAAGCATCTCT Reverse, GTGAGCATCCACCCAAATGAC
*ym1*	Forward, ATCTATGCCTTTGCTGGAATGC Reverse, TGAATGAATATCTGACGGTTCTGAG
